# Overexpression of alanine-glyoxylate aminotransferase 2 protects from asymmetric dimethylarginine-induced endothelial dysfunction and aortic remodeling

**DOI:** 10.1038/s41598-022-13169-2

**Published:** 2022-06-07

**Authors:** Roman N. Rodionov, Natalia Jarzebska, Dmitrii Burdin, Vladimir Todorov, Jens Martens-Lobenhoffer, Anja Hofmann, Anne Kolouschek, Nada Cordasic, Johannes Jacobi, Elena Rubets, Henning Morawietz, John F. O’Sullivan, Alexander G. Markov, Stefan R. Bornstein, Karl Hilgers, Renke Maas, Christian Pfluecke, YingJie Chen, Stefanie M. Bode-Böger, Christian P. M. Hugo, Bernd Hohenstein, Norbert Weiss

**Affiliations:** 1 University Center for Vascular Medicine and Department of Medicine III, Section Angiology, University Hospital Carl Gustav Carus, Technische Universität Dresden, Fetscherstrasse 74, 01307 Dresden, Germany; 2 Department of Anesthesiology and Critical Care Medicine, University Hospital Carl Gustav Carus, Technische Universität Dresden, 01307 Dresden, Germany; 3 Department of General Physiology, Saint-Petersburg State University, 199034 Saint-Petersburg, Russia; 4 Department of Medicine III, Section Nephrology, Technische Universität Dresden, 01307 Dresden, Germany; 5 Institute of Clinical Pharmacology, Otto-Von-Guericke University, 39120 Magdeburg, Germany; 6 Division of Vascular Endothelium and Microcirculation, Department of Medicine III, University Hospital Carl Gustav Carus, Technische Universität Dresden, 01307 Dresden, Germany; 7 Department of Nephrology and Hypertension, Universitätsklinikum Erlangen, 91054 Erlangen, Germany; 8 The University of Sydney, Charles Perkins Centre, Sydney, NSW Australia; 9 The University of Sydney, Heart Research Institute, Sydney, NSW Australia; 10 Department of Cardiology, Royal Prince Alfred Hospital, Camperdown, NSW Australia; 11 Department of Medicine III, University Hospital Carl Gustav Carus, Technische Universität Dresden, 01307 Dresden, Germany; 12 Institute of Experimental and Clinical Pharmacology and Toxicology, Friedrich-Alexander-Universität Erlangen-Nürnberg (FAU), 91054 Erlangen, Germany; 13 Department of Internal Medicine and Cardiology, Herzzentrum Dresden, University Clinic, Technische Universität Dresden, 01307 Dresden, Germany; 14 Cardiovascular Division, Department of Medicine, University of Minnesota, Minneapolis, MN 5455 USA

**Keywords:** Molecular biology, Cardiology

## Abstract

Elevated plasma concentrations of asymmetric dimethylarginine (ADMA) are associated with an increased risk of mortality and adverse cardiovascular outcomes. ADMA can be metabolized by dimethylarginine dimethylaminohydrolases (DDAHs) and by alanine-glyoxylate aminotransferase 2 (AGXT2). Deletion of DDAH1 in mice leads to elevation of ADMA in plasma and increase in blood pressure, while overexpression of human DDAH1 is associated with a lower plasma ADMA concentration and protective cardiovascular effects. The possible role of alternative metabolism of ADMA by AGXT2 remains to be elucidated. The goal of the current study was to test the hypothesis that transgenic overexpression of AGXT2 leads to lowering of plasma levels of ADMA and protection from vascular damage in the setting of DDAH1 deficiency. We generated transgenic mice (TG) with ubiquitous overexpression of *AGXT2*. qPCR and Western Blot confirmed the expression of the transgene. Systemic ADMA levels were decreased by 15% in TG mice. In comparison with wild type animals plasma levels of asymmetric dimethylguanidino valeric acid (ADGV), the AGXT2 associated metabolite of ADMA, were six times higher. We crossed AGXT2 TG mice with DDAH1 knockout mice and observed that upregulation of AGXT2 lowers plasma ADMA and pulse pressure and protects the mice from endothelial dysfunction and adverse aortic remodeling. Upregulation of AGXT2 led to lowering of ADMA levels and protection from ADMA-induced vascular damage in the setting of DDAH1 deficiency*.* This is especially important, because all the efforts to develop pharmacological ADMA-lowering interventions by means of upregulation of DDAHs have been unsuccessful.

## Introduction

Elevated plasma concentrations of asymmetric dimethylarginine (ADMA) are independently associated with adverse cardiovascular effect^1^ increased mortality, and adverse cardiovascular outcomes^2^. In vitro experiments and mouse models of disrupted^3,4^ or augmented^5,6^ ADMA metabolism suggest a causal role. The ADMA action as an inhibitor of nitric oxide synthase^7,8^ is considered a major, but possibly not the only, underlying patho-mechanism. This has opened the quest for therapeutic approaches to lower plasma ADMA, with a focus on metabolism^9^ and transport as possible targets^10^. The major metabolic route responsible for ADMA breakdown is its hydrolysis by dimethylarginine dimethylaminohydrolases 1 and 2 (DDAH1 and DDAH2) with formation of citrulline and dimethylamine. The relative contribution of DDAH2 towards ADMA clearance is still a matter of controversy^4,8,11,12^. The DDAH-ADMA pathway has been shown to play an important role in vascular homeostasis in vivo^13^*.* Heterozygous *Ddah1* knockout mice demonstrated increased ADMA levels, elevated blood pressure and impairment of NO-related endothelial function^3^. In contrast, overexpression of either DDAH1 or DDAH2 in transgenic mice led to decreased plasma ADMA levels and increased NO bioavailability^11,14^.

Several studies suggested that the increase of ADMA in cardiovascular and metabolic diseases is caused by downregulation of DDAHs at the level of expression or activity^15–17^. Manipulations of activity of ADMA-lowering enzymes might be a promising strategy for treatment of cardiovascular diseases. To our best knowledge, multiple attempts to develop small molecules upregulating DDAHs have failed, potentially because these enzymes are very small and possibly do not have sites for allosteric regulation^18–20^.

Although the DDAHs are thought to represent the main and the best characterized metabolic pathway for the elimination of ADMA, the enzyme alanine:glyoxylate aminotransferase 2 (AGXT2) has also been suggested to play an important role in ADMA catabolism. AGXT2 is a mitochondrial aminotransferase, expressed primarily in kidney and liver, catalyzing transamination of ADMA as the amino donor with formation of asymmetric α-keto-δ-(*N*,*N*-dimethylguanidino)valeric acid (ADGV). Downregulation of AGXT2 leads to elevation of systemic ADMA levels^21,22^. We have previously demonstrated that overexpression of AGXT2 by adenoviral vectors lowers ADMA in vivo^23^. However, the transient character of adenoviral overexpression did not allow estimation of the long term physiological consequences of AGXT2 upregulation.

AGXT2 might be an alternative target for ADMA-lowering interventions. The goal of the current study was to test the hypothesis that upregulation of AGXT2 can protect from ADMA-induced vascular damage in the setting of DDAH1-deficiency. To test this hypothesis we generated and characterized AGXT2 transgenic mice and determined, whether this transgene can protect Ddah1 knockout mice from ADMA elevation, endothelial dysfunction and aortic remodeling.

## Materials and methods

### Generation of AGXT2 transgenic mice

All animal experiments were approved by local authorities and were performed according to the guidelines from Directive 2010/63/EU of the European Parliament on the protection of animals used for scientific purposes. The study is reported in accordance with ARRIVE guidelines.

C57BL/6J mice (The Jackson Laboratory, Bar Harbor, ME, USA) were used to create the AGXT2 transgenic line (AGXT2 TG). Protocols were approved by the animal welfare committee of the Technische Universität Dresden, Germany. Mice were maintained on a 12-h light/dark cycle and provided with standard chow and water ad libitum. A human AGXT2 transgenic construct, containing human AGXT2 coupled with a FLAG epitope on C-terminus (GenBank accession number KU847977), a CAG promoter^24^ and RNA processing signals from SV40 polyA was synthesized by GenScript (www.genscript.com). This 3761-bp sequence was cloned into the EcoRV site of the pUC57 vector. The resulting construct was digested with ScaI/SalI/BglII. After agarose gel electrophoresis, the DNA fragment containing the targeting construct was excised as a ScaI-SalI fragment, purified and used for the generation of transgenic mice. Fertilized eggs were harvested from superovulated C57BL/6J mice for pronuclear injection of 5 µL of purified DNA. Embryos were transferred into the oviducts of pseudopregnant foster mothers. Genomic DNA was isolated from tail biopsies at 3–4 weeks of age using a DNeasy kit (Qiagen, Hilden, Germany) and analyzed by PCR to identify the presence of the transgene. PCR analysis was performed using transgene-specific oligonucleotide primers (forward 5′-GTTGGCAGAGGCAGCATT and reverse 5′-GTCGTCATCCTTGTAATCCTTAGC). Transgenic mice were compared with age-, weight-, and sex-matched wild type littermates.

### Crossing of AGXT2 TG mice with DDAH1 KO mice

*Ddah1* knockout mice were bred, genotyped and housed as previously described^4^. Heterozygous *Ddah1* knockout mice (DDAH1 KO) were bred with heterozygous AGXT2 TG mice to generate homozygous DDAH1 KO littermates with and without AGXT2 transgene.

### Collection of plasma and tissues

Mice were anesthetized with ketamine/xylazine solution (15 mg/mL ketamine, 1 mg/mL xylazine, dose 0.1 mL/10 g body weight) and blood was collected by cardiac puncture into ethylenediaminetetraacetic acid (EDTA)–containing tubes (final concentration of EDTA: 5 mmol/L). Plasma was separated by centrifugation and stored at − 80 °C. Tissue samples of aorta, brain, heart, kidney, liver, lung, skeletal muscle and spleen were collected, immediately afterwards flash frozen in liquid nitrogen and stored at − 80 °C until further analysis.

### Measurement of ADMA, SDMA, ADGV and creatinine in plasma and urine

Plasma and urine levels of ADMA were measured by isotope dilution liquid chromatography-tandem mass spectrometry (LC–MS–MS) as described previously^25^. ADGV levels in plasma were also determined by LC–MS/MS, applying the procedure specific for this analyte^26^. Creatinine was measured by high-performance liquid chromatography as described previously^27^.

### Blood pressure measurements

Blood pressure was measured in 16 week old mice using an intra-arterial telemetric transmitter system (PhysioTel^®^, TA11PAC10, Data Sciences International (DSI), New Brighton, MN, USA) as described in Ref.^28^. Briefly, a catheter connected to the blood pressure sensor was placed in the left common carotid artery under general isoflurane anesthesia and the transmitter was implanted subcutaneously over the abdominal area. Mean arterial pressure was detected and recorded continuously for 4 weeks by the Dataquest A.R.T. TM System (Data Sciences International (DSI), New Brighton, MN, USA). Mean blood pressure values or heart rates were calculated by the Dataquest^®^ software. The protocol was approved by the animal welfare committee.

### Real-time polymerase chain reaction

Messenger RNA (mRNA) levels of human AGXT2 transgene, mouse AGXT2, total (endogenous + transgenic) AGXT2 genes, DDAH1 and DDAH2 genes were determined by real-time reverse transcription-polymerase chain reaction (RT-PCR). The RNA isolation from tissues was carried out using the RNeasy RNA extraction kit (Qiagen, Hilden, Germany). Reversed transcription of the isolated RNA to cDNA was achieved by applying the High Capacity cDNA Reverse Transcription Kit (Applied Biosystems, Foster City, USA). All kits were applied according to the manufacturer’s instructions. The amplification of the cDNA templates for quantification was carried out using the Maxima SYBR Green/ROX Master Mix (Thermo Fisher Scientific, Dreieich, Germany). DNA was first denaturated for 10 min at 95 °C, followed by 40 cycles of denaturation for 15 s at 95 °C and annealing and extending for 60 s at 60 °C. Data were analyzed using Applied Biosystems 7500 software (version 2.0.5, Applied Biosystems, Foster City, CA, USA) and expressed as a ratio to levels of hypoxantin-guanin-phosphoribosyltransferase (HPRT) mRNA. The primer pairs used in the real-time RT-PCR for amplification of the respective cDNA sequences were designed using the Sigma Oligoarchitect primer tool (www.oligoarchitect.com). The selected primer pair sequences for mouse AGXT2 were forward 5′-CAGGGAGGAAGGCGAGAATG and reverse 5′-CTTGGCAGCAGTCTGTAGCA; human transgenic AGXT2 forward 5′-GTTGGCAGAGGCAGCATT and reverse 5′-GTCGTCATCCTTGTAATCCTTAGC; total (transgenic + endogenous) AGXT2 forward 5′-TGGGCTCTCACTTCTGGG and reverse 5′-CACCTCAAGCACAGCAGAT; mouse DDAH1 forward 5′-CTACGCAGTCTCTACAGT and reverse 5′-TCATAACGATGGTCACTCA; mouse DDAH2 forward 5′-AAAGCAGTCAGGGCAATG and reverse 5′-CCAGGACGCAGAAAGAGA, mouse HPRT forward 5′-CTTTGCTGACCTGCTGGATTAC and reverse 5′-GGACCTCTCGAAGTGTTGGAT. The primer oligomers were synthesized by Biomers (Ulm, Germany).

### Western blot

Tissue samples of mice were homogenized in ice-cold 0.2% SDS solution containing a protease inhibitor mixture (Mini-complete Protease Inhibitor Cocktail Tablets; Roche Diagnostics-Applied Science, Mannheim, Germany). Protein concentrations were determined using a standard BCA protein assay (Rockford, IL, USA) according to manufacturer’s instructions.

For immunoblot analysis, tissue homogenates (15 μg of total protein) from aorta, brain, heart, kidney, liver, lung, skeletal muscle and spleen of mice were prepared and diluted with Laemmli buffer (62 mM Tris–HCl pH 6.8, 2% SDS, 10% glycerol, 0.01% bromophenol blue, and 0.4 mM dithiothreitol). After incubation at 95 °C for 5 min, proteins were separated by SDS–PAGE under reducing conditions on 10% polyacrylamide gels and transferred to nitrocellulose membranes (Protran Nitrocellulose Transfer Membrane; Whatman, Dassel, Germany) using a tank blotting system from Bio-Rad (Munich, Germany). The Western Blot analysis was performed as described in Ref.^29^. Membranes were stained by Ponceau reagent (Sigma-Aldrich, St. Louis, MI, USA) to control for the protein transfer and then blocked in 5% milk for 1 h at 37 °C. For AGXT2-FLAG detection membranes were incubated overnight at 4 °C with a 1:500 mouse monoclonal anti-FLAG antibody (Sigma-Aldrich, St. Louis, MI, USA, Catalog #F3165), washed three times in TBST (50 mM Tris–HCl pH 7.5, 150 mM NaCl, 0.1% Tween 20) and then incubated with a 1:10,000 horseradish peroxidase-conjugated goat anti-mouse antibody (BD Biosciences, San Jose, CA, USA) for 2 h at room temperature (RT). Immunoreactive bands were visualized using Roche Lumi-Light Western Blotting Substrate (Roche Diagnostics, Mannheim, Germany). To control for sample loading, membranes were incubated with 1:500 polyclonal rabbit anti-GAPDH antibodies (Trevigen, Gaithersburg, MD, USA, Catalog # 2275-pc-100) for 1 h at RT, washed three times in TBST (50 mM Tris–HCl pH 7.5, 150 mM NaCl, 0.1% Tween 20) and then incubated with a 1:10,000 horseradish peroxidase-conjugated goat anti-rabbit antibody (The Jackson Laboratory, Bar Harbor, ME, USA) for 2 h at RT. Blotted proteins were detected using a PeqLab Fusion Fx7 Imaging System (PeqLab, Erlangen, Germany).

### Vasomotor function studies ex vivo

Mice were anesthetized with ketamine (100 mg/kg i.p.) and xylazine (10 mg/kg i.p.) followed by dissection of the thoracic aorta. Aortas were placed in Krebs’ buffer, connective tissue was removed, and vessels were cut into rings (length 3–4 mm). Aortic rings were placed into individual wells of 48-well cell-culture dishes containing 0.5 mL Dulbecco’s modified Eagle’s medium containing 5 mmol/L glucose, 120 U/mL penicillin, 120 g/mL streptomycin and 50 g/mL polymyxin B. Vascular rings were connected to a force transducer at a final tension of 0.5 g to measure isometric tension in an organ bath containing Krebs’ solution maintained at 37 °C as previously described^30,31^. At the beginning of the experiment dose–response curves to phenylephrine (PE) were obtained. After resting tone was reestablished, the vessels were precontracted to 60% of the maximal response to PE. Afterwards, relaxation dose–response curves were generated by cumulative addition of the endothelium-dependent vasodilator acetylcholine (ACh) or the endothelium-independent vasodilator sodium nitroprusside (SNP). The percent contraction in response to SNP and ACh was calculated in comparison to baseline.

### Cell culture studies

Primary aortic cells were isolated from murine aortas as described previously^32^. Briefly, two male mice were anesthetized with an intraperitoneal injection of 0.3–0.4 mL of pentobarbital sodium (10 mg/mL) per mouse. The aorta was dissected from the aortic arch to the abdominal aorta, and immersed in DMEM with 20% FBS containing 1000 U/mL of heparin. The fat and connecting tissues were rapidly removed with fine forceps under a stereoscopic microscope. A 24-gauge cannula was then inserted into the proximal portion of the aorta. After ligation at the site with a silk thread, the lumen was briefly washed with serum-free DMEM. The other side was sutured and the aorta was filled with collagenase type II solution (2 mg/mL, dissolved in serum-free DMEM). After incubation for 45 min at 37 °C, endothelial cells were removed from the aorta by flushing with 5 mL of DMEM containing 20% FBS. Endothelial cells were collected by centrifugation at 1200 rpm for 5 min. The pelleted cells were gently resuspended by pipette with 2 mL of 20% FBS-DMEM and cultured in a 35 mm collagen type I-coated dish. To remove smooth muscle cells, after 2 h incubation at 37 °C the medium was removed, the cells were washed with warmed PBS, and medium G (20% FBS, 100 U/mL penicillin-G, 100 μg/mL streptomycin, 2 mM l-glutamine, 1 × non-essential amino acids, 1 × so-1 × sodium pyruvate, 25 mM HEPES (pH 7.0–7.6), 100 μg/mL heparin, 100 μg/mL ECGS, and DMEM) was added. The cells were grown for 1 week, and used for subsequent immunocytochemistry.

### Immunocytochemistry

To analyze human AGXT2 transgene expression cell slides with primary aortic cells were fixed with 1:1 acetone-methanol solution for 10 min at 4 °C, washed 3 × 2 min with ice-cold PBS and blocked with Dako Protein Blocking solution for 20 min at room temperature. Afterwards, chamber slides were incubated with 1:100 rabbit polyclonal anti-FLAG antibodies (Sigma-Aldrich, St. Louis, MI, USA, Catalog #7425) for 2 h at 37 °C, washed 3 × 2 min with PBS and subsequently incubated with 1:250 anti-rabbit antibodies coupled with fluorescence dye (The Jackson Laboratory, Bar Harbor, ME, USA) at room temperature for 1 h. Finally, slides were washed 3 × 2 min with PBS, stained with 1:1000 DAPI and mounted with Moviol. To demonstrate the expression of human AGXT2-FLAG transgene in endothelial cells 1:100 rat anti-CD31 antibodies (Biolegend, San Diego, CA, USA, Catalog # 102401) (as marker of endothelial cells^33^), and 1:250 anti-rat secondary antibodies (The Jackson Laboratory, Bar Harbor, ME, USA) were used. Double staining of CD31 and FLAG-tagged transgene was performed in the same way.

### Histochemistry

Immediately after isolation heart samples were fixed in cold 4% paraformaldehyde diluted in phosphate-buffered saline at 4 °C and processed for paraffin embedding, cross-sectioned to obtain 4 µm-thick sections and mounted on glass slides. Tissue sections were deparaffinized in xylene 3 × 5 min and rehydrated in descending concentrations of ethanol (100%, 96%, 70% and 40%, 2 min each). Picrosirius red staining for collagen I and III was performed on rehydrated sections using 1% Sirius Red/Fast Green solution for 30 min at room temperature. Staining for elastin was performed on rehydrated sections using resorcin-fuchsine solution for 30 min at room temperature. The slides were then washed in distilled water for 2 min, immersed in 80% ethanol for 5–10 min and put in distilled water. The sections were then dehydrated in ascending concentrations of ethanol, cleared in xylene 3 × 5 min and mounted with DePeX mounting medium. The morphometric analysis was performed in ImageJ (National Institute of Health).

### Statistical analysis

Statistical analysis was performed using GraphPad Prism 9.0.0. Comparisons between the groups were performed by one-way ANOVA (with factor groups) followed by Tukey multiple comparison test or two-way ANOVA (with a repeated measures factor time and factor group) followed by Sidak multiple comparison test. Statistical significance was defined as a P value < 0.05. Values are reported as mean ± standard error of the mean. N number and the name of the specific statistical test performed are provided in the figure legends.

## Results

### Phenotype of AGXT2 transgenic mice

**Figure 1:**
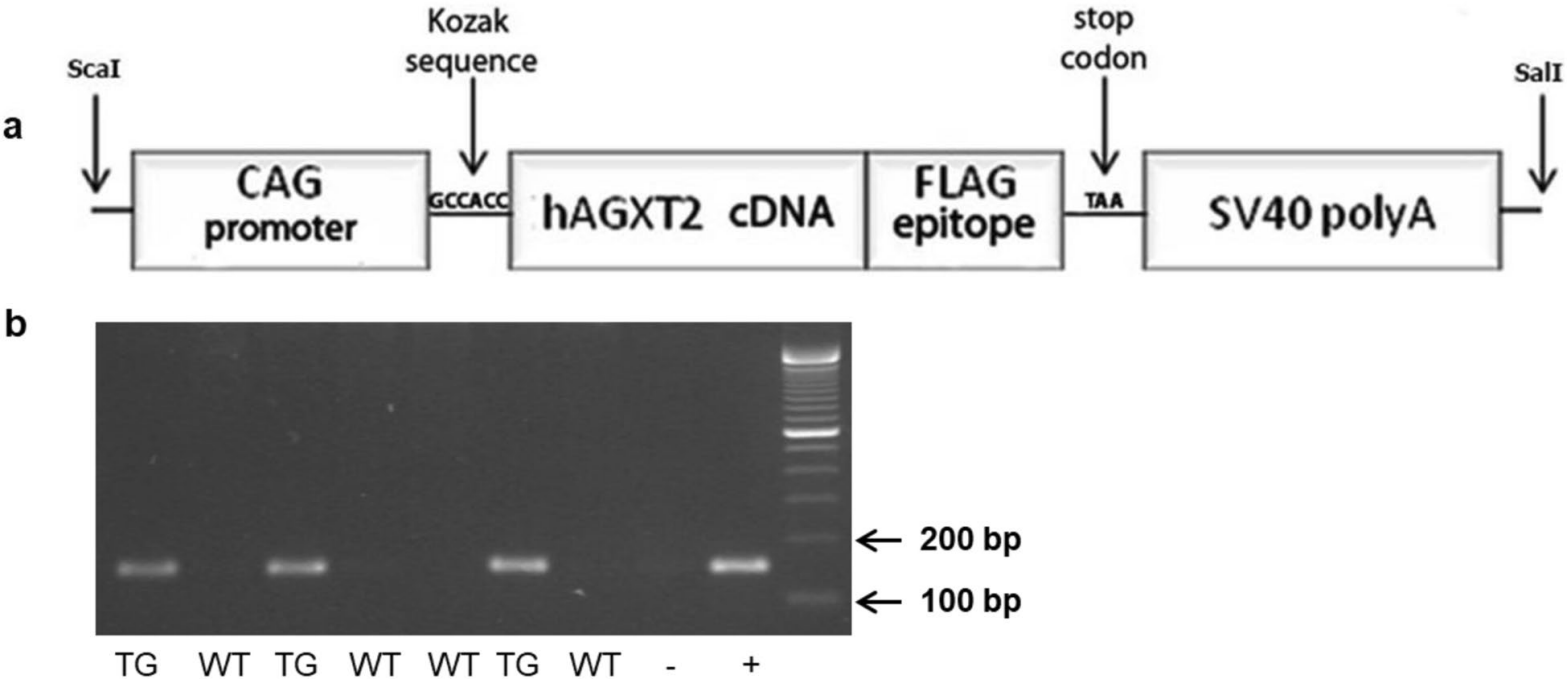
Generation of AGXT2 TG mice. (**a**) The construct used for the generation of AGXT2 transgenic mice contains a 3.3-kb fragment from the CAG promoter, a 1.5-kb human AGXT2 open reading frame coupled with a FLAG epitope and a SV-40 polyadenylation sequence. Restriction sites used for cloning are indicated by arrows. (**b**) PCR analysis detects 150 bp-PCR products containing sequences of the AGXT2 transgene. − negative control (empty pUC57 vector); + positive control (pUC57 vector containing hAGXT2 sequence).

We successfully generated the AGXT2 transgenic mice. PCR analysis revealed the 150-bp PCR fragments of the transgene in AGXT2 TG mice, whereas in WT mice these fragments were absent (Fig. 1). Transgenic mice did not differ from control littermates in general appearance. They developed normally and were fertile. Transgenic offspring was obtained in a Mendelian ratio. Detailed necropsies of 15 mice (7 male/8 female) aged 12 weeks revealed no anatomic abnormalities. There was no significant difference in the systolic blood pressure between the TG and WT mice measured by the telemetric transmitter system method (Supplementary Data). Aortas from transgenic mice had improved endothelial response to acetylcholine as compared to wild type littermates (Supplementary Data).

### Distribution of AGXT2 transgene in various tissues

**Figure 2:**
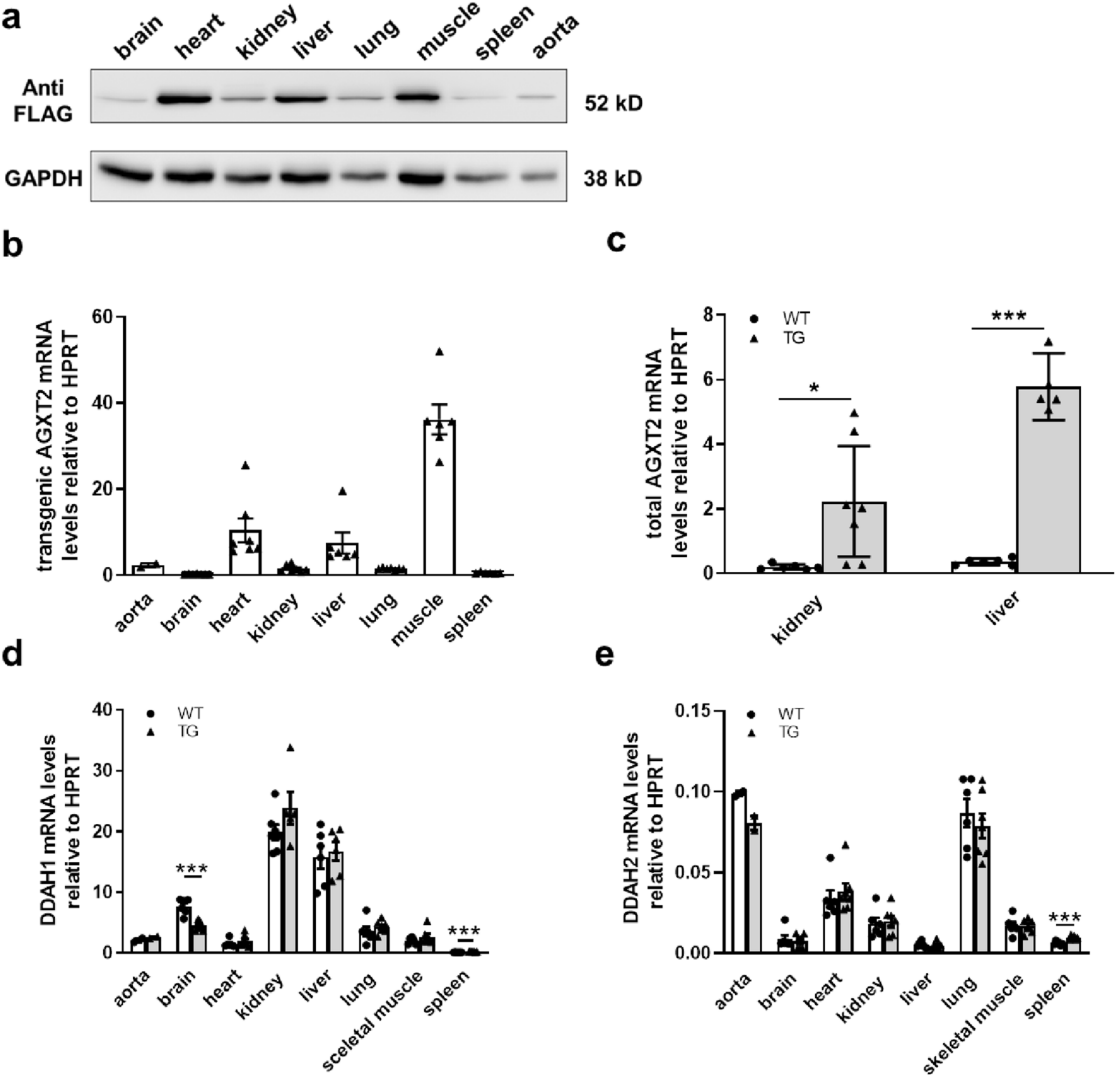
(**a**) Distribution of AGXT2 transgene in different tissues of TG mice, detected by Western blotting. (**b**) Distribution of AGXT2 transgene in various tissues of TG mice, detected by qPCR, n = 5–7 per group. (**c**) Expression of total (endogenous and transgenic) AGXT2 in kidney and liver of transgenic and wild-type mice. (**d**) Expression of DDAH1 and DDAH2 (**e**) in various tissues of TG mice, detected by qPCR. *p < 0.05; ***p < 0.001; One-way ANOVA followed by Tukey’s multiple comparison test (**c**–**e**). N = 6–7 (**a**), 5–7 (**b**–**d**). Cohen’s d = (**c**) 1.7 (kidney) and 10.2 (liver), (**d**) 3.4 (brain) and 2.2 (spleen); (**e**) 2.3 (spleen).

The AGXT2 transgene was detected by qPCR in all tissues examined. The maximum amount of human AGXT2 was revealed by Western Blot in heart, liver and skeletal muscle (Fig. 2a). These results were consistent with results obtained by RT-PCR (Fig. 2b). Total expression of AGXT2 in kidney and liver (the only organs expressing endogenous AGXT2) from AGXT2 transgenic mice were ~ 10 times higher than in WT mice (Fig. 2c). There were no major differences in DDAH1 and DDAH2 expression levels between transgenic and wild-type animals (Fig. 2d,e).

### Effect of transgenic AGXT2 overexpression on ADMA and ADGV plasma, urine and tissue levels

**Figure 3:**
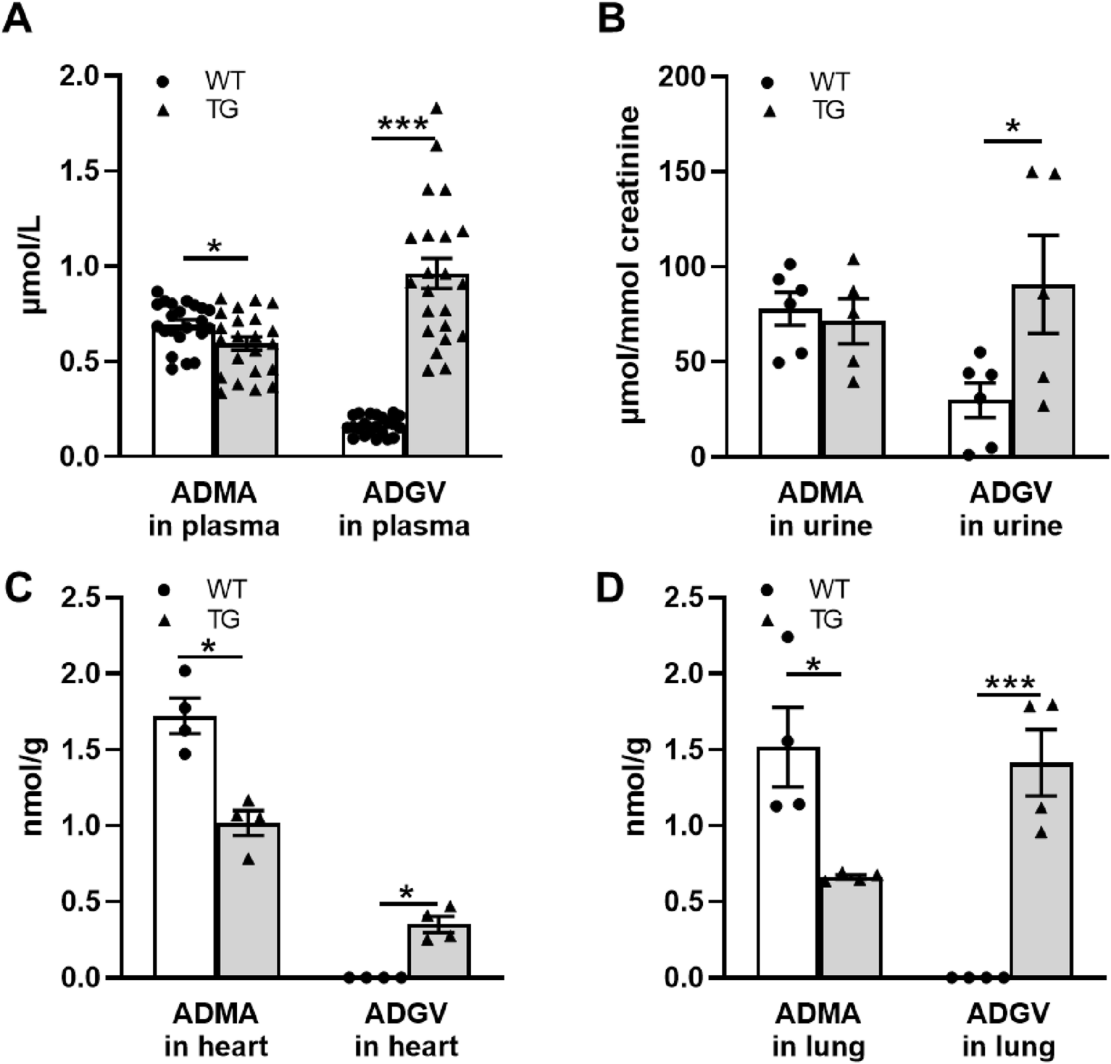
Biochemical analysis of metabolites in wild-type (WT) and AGXT2 transgenic (TG) mice. (**a**) Plasma levels of ADMA and ADGV (**b**) Urine levels of ADMA and ADGV (**c**) Content of ADMA and ADGV in the heart (**d**) Content of ADMA and ADGV in the lung. *p < 0.05; ***p < 0.001. One-way ANOVA followed by Tukey’s multiple comparison test. N = 22 (**a**), 5–6 (**b**), 4 (**c**,**d**). Cohen’s d = (**a**) 0.7 (ADMA) and 2.9 (ADGV), (**b**) 1.4, (**c**) 3.5 (ADMA) and 4.7 (ADGV); (**d**) 2.4 (ADMA) and 4.6 (ADGV).

Biochemical analysis of mice overexpressing AGXT2 demonstrated lower plasma ADMA levels in these mice (13% lower versus WT mice; Fig. 3a), whereas ADGV levels in both plasma and urine of TG mice were 4 times higher (Fig. 3a,b) in comparison with controls. Tissue content of ADMA in hearts and lungs of AGXT2 transgenic mice were 50% lower in comparison with WT controls. The tissue levels of ADGV in hearts and lungs and of AGXT2 TG mice were significantly higher than in controls (Fig. 3c,d).

### Immunofluorescence analysis of AGXT2 TG aortas

**Figure 4:**
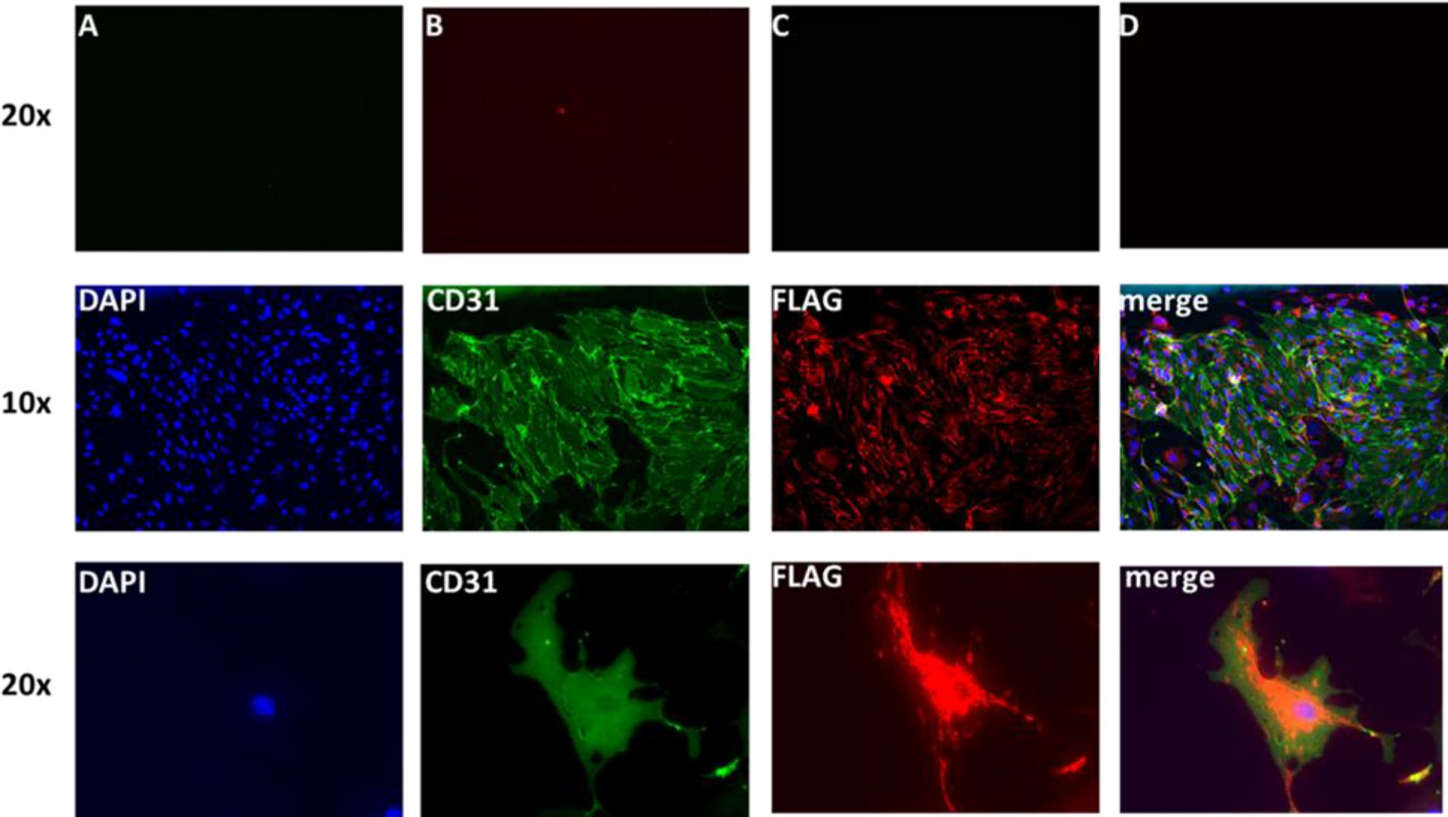
Immunofluorescence staining of aortic endothelial cells isolated from aorta of AGXT2 TG mice, showing the expression of transgene in endothelial cells (CD31 positive). First row: (**A**) CD31 negative control in green channel, (**B**) CD31 negative control in red channel, (**C**) FLAG negative control in green channel, (**D**) FLAG negative control in red channel.

To further confirm the expression of AGXT2 transgene in endothelial cells in the aorta we used double immunofluorescence staining. The AGXT2 transgene was detected in primary cells from aortas, which also expressed CD31 as an endothelial marker^28^ (Fig. 4).

### Rescue of phenotype of DDAH1 KO mice by transgenic overexpression of AGXT2

**Figure 5:**
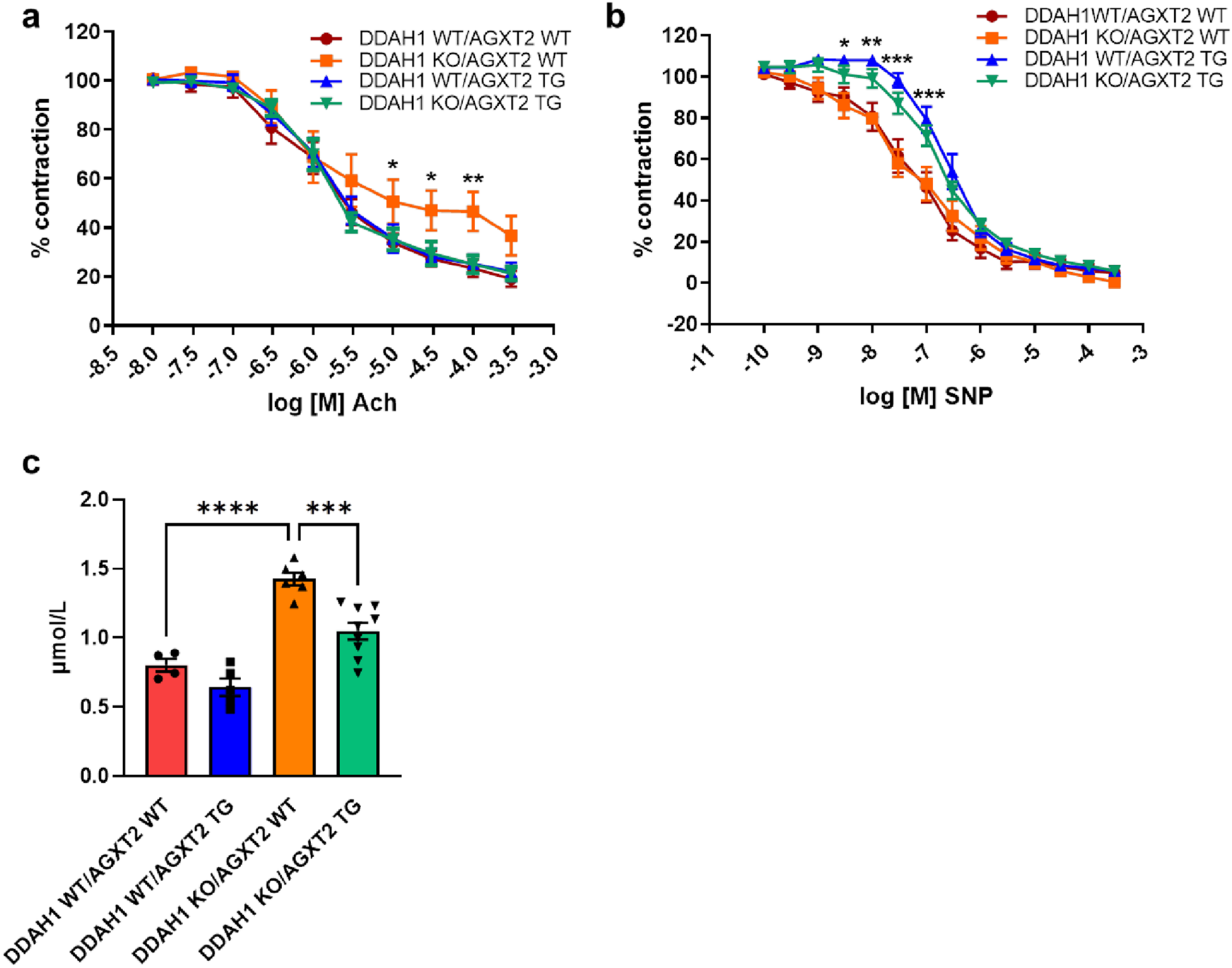
(**a**) Endothelium-dependent and (**b**) -independent vasodilation and (**c**) ADMA plasma levels in DDAH1 KO/AGXT2 WT, DDAH1 KO/AGXT2 WT, DDAH1 WT/AGXT2 TG and DDAH1 KO/AGXT2 TG mice. *Ach* acetylcholine, *SNP* sodium nitroprusside. Two-way repeated-measures ANOVA followed by Sidak multiple comparison test (**a**,**b**), *p < 0.05; **p < 0.01; p < 0.001 DDAH1 KO/AGXT2 WT vs. DDAH1 KO/AGXT2 TG. One-way ANOVA followed by Tukey’s multiple comparison test (**c**), ***p < 0.001; ****p < 0.0001. N = 8–15 (**a**,**b**), 4–9 (**c**). Cohen’s d = (**c**) 4.6 (DDAH1 WT/AGXT2 WT vs DDAH1 KO/AGXT2 WT) and 2.5 (DDAH1 KO/AGXT2 WT vs DDAH1 KO/AGXT2 TG).

**Figure 6:**
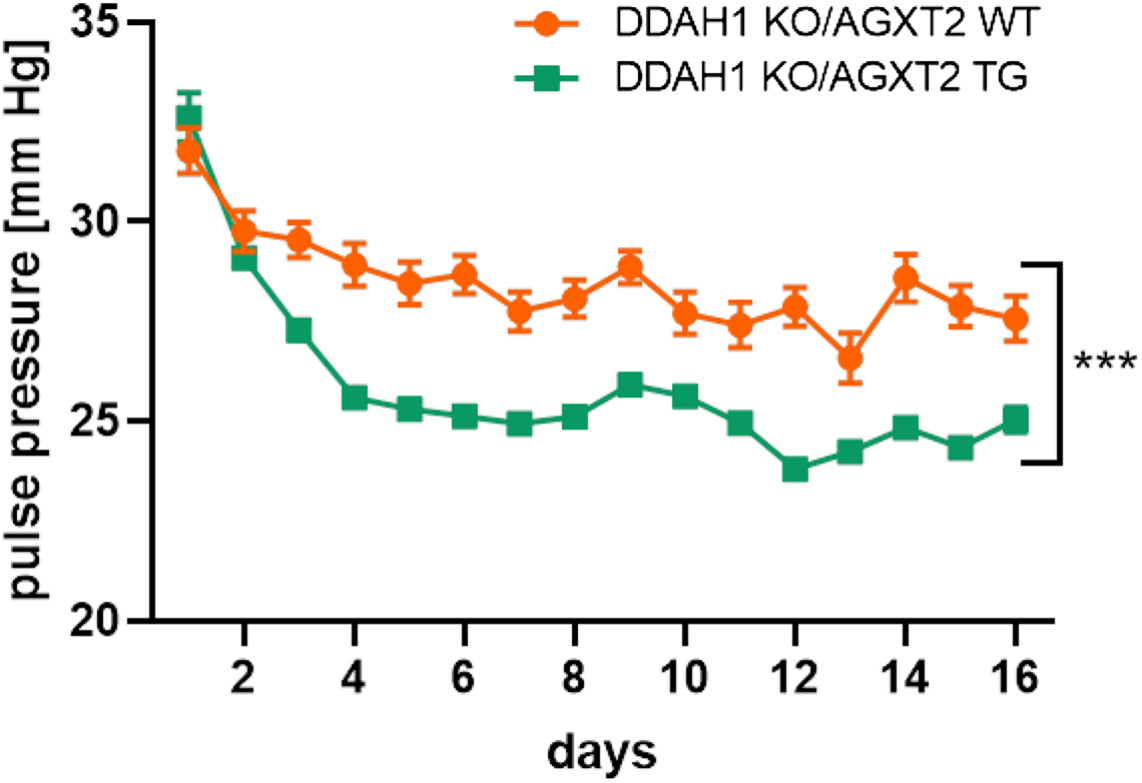
Pulse pressure in DDAH1 KO/AGXT2 WT and DDAH1 KO/AGXT2 TG mice. The recordings in the first 7–8 days can be influenced by stress after implantation of the sensor and the transmitter. Repeated-measures ANOVA followed by Sidak multiple comparison test, ***p < 0.001. N = 6–11.

**Figure 7:**
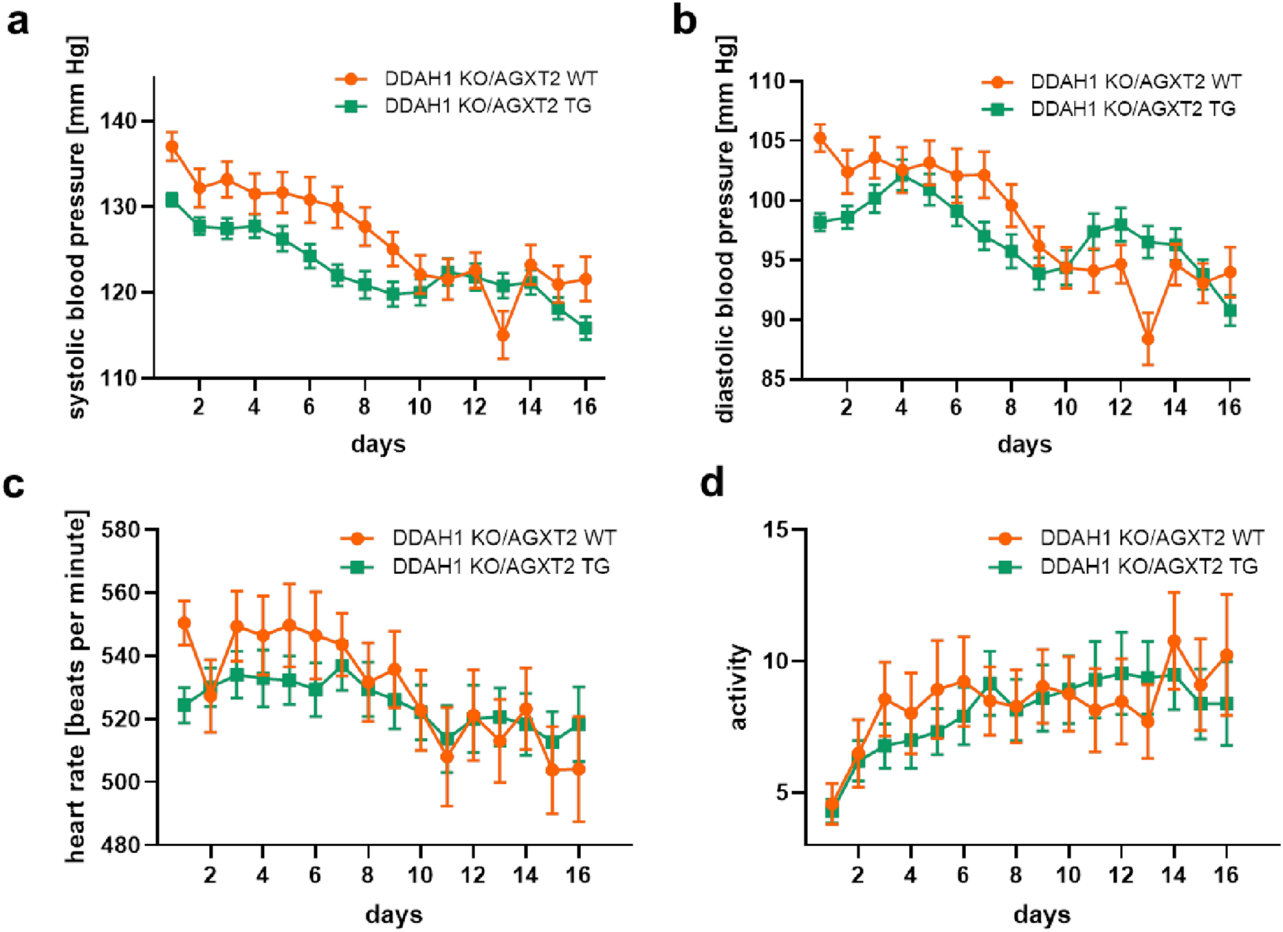
Systolic (**a**) and diastolic (**b**) blood pressure, heart rate (**c**) and activity (**d**) in DDAH1 KO/AGXT2 WT and DDAH1 KO/AGXT2 TG mice. Repeated-measures ANOVA followed by Sidak multiple comparison test. N = 6–13.

**Figure 8:**
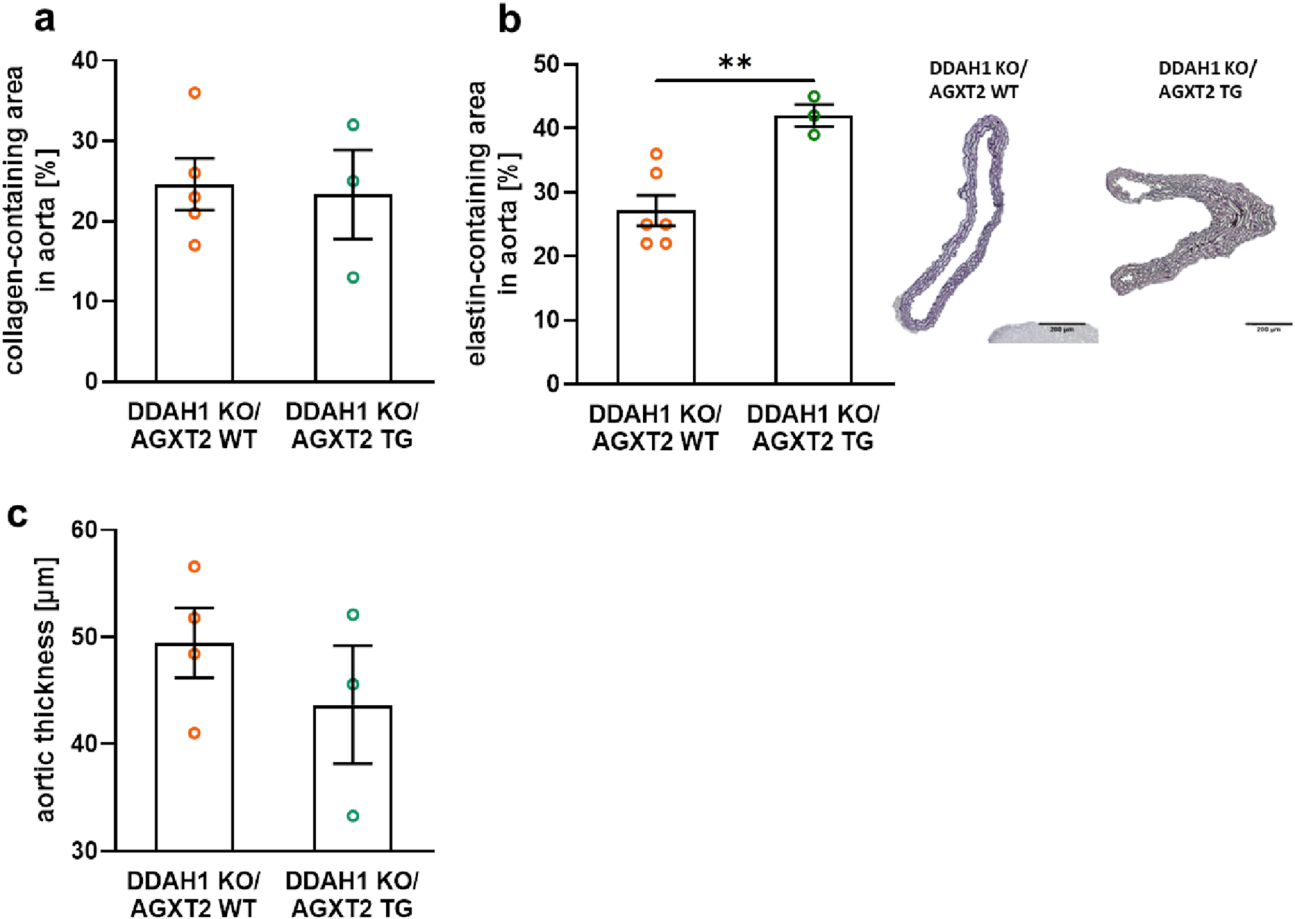
Collagen- (**a**) and elastin-containing areas in aorta (**b**) and aortic thickness (**c**) in DDAH1 KO/AGXT2 WT and DDAH1 KO/AGXT2 TG mice. One-way ANOVA flowed by Tukey’s multiple comparison test, **p < 0.01. N = 3–6. Cohen’s d = 3.1 (elastin).

Aortas from DDAH1 KO mice crossed with AGXT2 TG mice demonstrated significantly improved endothelium-dependent vasodilatation in comparison with DDAH1 KO mice (Fig. 5a). Endothelium-independent vasodilatation was diminished at low concentrations of sodium nitroprusside in the AGXT2 TG group compared to the AGXT2 TG crossed with DDAH1 KO mice (Fig. 5b). Plasma ADMA levels in DDAH KO mice crossed with AGXT2 TG mice were significantly lower than in DDAH1 KO mice (Fig. 5c). AGXT2 TG mice also had substantially reduced pulse pressure as compared to the DDAH1 KO mice crossed with AGXT2 TG animals (Fig. 6). There were no significant differences in systolic and diastolic blood pressure, heart rate or activity of the animals (Fig. 7a–d). In line with the difference in pulse pressure, the elastin content was increased in AGXT2 TG crossed with DDAH1 KO mice as compared to the DDAH1 KO animals (Fig. 8).

## Discussion

In the current manuscript we report the successful generation of transgenic mice with global overexpression of human AGXT2. The mice were viable, with normal growth development and fertility. The transgene was present in all tissues examined and did not influence the expression of DDAH1 or DDAH2 in the majority of tissues. The significant decrease in the plasma and tissue ADMA levels and increase in ADGV levels (product of ADMA metabolism by AGXT2) in comparison to the wild type animals demonstrated the physiological importance of AGXT2 in maintaining homeostatic levels of endogenous methylarginines. The observation that the decrease in plasma ADMA levels was less pronounced, than the associated with it increase in ADGV levels points to the vital importance of maintaining homeostatic ADMA concentrations and tight regulation of its levels.

In the second part of the manuscript, we were able to demonstrate that overexpression of AGXT2 restores physiological levels of ADMA and protects from endothelial dysfunction in DDAH1 deficiency. The mechanism responsible for the impaired response to the low concentrations of the endothelium-independent vasodilator SNP observed in the AGXT2 TG mice irrespective of DDAH1 is not clear. It might indicate a slight desensitization of the signal transduction pathway downstream of NO, as a possible compensatory effect for chronically decreased intracellular ADMA levels. Furthermore, overexpression of AGXT2 in DDAH1 KO mice led to a decrease in pulse blood pressure without affecting physical activity and heart rate. Interestingly, the amount of elastic fibers in aortas of DDAH1 KO mice was lower than in aortas of the DDAH1 KO/AGXT2 TG littermates. This suggests that a decrease in pulse pressure in the DDAH1 KO/AGXT2 TG compared to the DDAH1 KO littermates could be explained by the protective effects of AGXT2 overexpression and ADMA lowering on aortic remodeling.

Generation of the DDAH1 transgenic mice (DDAH1 TG) was a major breakthrough in cardiovascular research. It demonstrated that upregulation of DDAH1 lowers plasma and tissue ADMA levels and increases basal and stimulated NO production. It also protected from experimental atherosclerosis, hind limb ischemia, myocardial ischemia–reperfusion injury, from vascular hypertrophy and stiffening in the setting of hypertension and hyperhomocysteinemia and from graft coronary artery disease^5,6,14,34–40^. Given the multiple cardiovascular protective effects of DDAH1 overexpression in mice mentioned above, therapeutic upregulation of DDAH1 may provide major benefits to patients with high cardiovascular risk. Several screens for small molecules modulating DDAH1 activity yielded multiple specific DDAH1 inhibitors, but unfortunately no promising DDAH1 activators^18–20,41^. One potential explanation might be that DDAH1 has a relatively small size and does not require cofactors. Therefore, it is unlikely that the enzyme has endogenous allosteric regulatory mechanisms, which could be therapeutically upregulated. Lack of DDAH1 activators might have affected the interest to target ADMA as a cardiovascular risk factor. In contrast to DDAHs, AGXT2 is larger and requires a cofactor, which increases the probability that this enzyme could be allosterically upregulated by small molecules (i.e. serve as a target for pharmacological interventions). Our study is therefore a key “proof of principle” investigation analysing the feasibility of therapeutic lowering of ADMA by targeting AGXT2. At least in our model, chronic upregulation of AGXT2 activity lowered systemic ADMA levels and protected from ADMA-induced vascular injury without any obvious phenotypical abnormalities. Further investigations of physiological consequences of chronic AGXT2 upregulation in other models of cardiovascular, metabolic and renal injury are needed to further assess the potential of AGXT2 upregulation as a therapeutic ADMA-lowering intervention. Our newly generated AGXT2 TG mouse line provides a valuable research tool for this purpose. However, it has to be emphasized that AGXT2 has multiple substrates, which raises a concern about potential side effects of the chronic increase of its enzymatic activity.

While the multiple protective cardiovascular phenotypes of DDAH1 transgenic mice are strongly supportive for the causative role of ADMA in cardiovascular injury, a possibility still exists that at least some of the beneficial cardiovascular effects of DDAH1 upregulation might be ADMA-independent. Indeed, in last few years the non-enzymatic effects of DDAH1 have been investigated. The group of Tokuo and colleagues showed that DDAH1 can bind to tumor suppressor neurofibromin 1 (NF1), which is a negative regulator of the Ras pathway increasing its phosphorylation by protein kinase A^42^. NF1 is known to regulate the migration and proliferation of vascular smooth muscle cells^43^. Therefore, there is an intriguing possibility that the altered NF1 function may be at least partially responsible for the vascular phenotype observed in the DDAH1 transgenic mice. The existence of ADMA-independent functions of DDAH1 is also supported by the study showing that upregulation of DDAH1 with mutated active site was able to influence the growth of glioma xenografts, while the total DDAH activity in the cells lysates was no altered^44^. What is more, downregulation of DDAH1 in a mouse model of bleomycin-induced lung fibrosis decreased collagen production by fibroblasts in an ADMA-independent manner^45^. Generation of the AGXT2 TG mice, which is described in the current manuscript, provides a valuable tool for lowering ADMA in vivo by a DDAH1-independent mechanism and therefore allows discrimination between protective effects mediated by lowering ADMA and other potentially beneficial mechanism implicating the non-enzymatic functions of DDAH1. Our current data from DDAH1 KO mice overexpressing AGXT2 showed for the first time that ADMA-lowering can protect from vascular injury even in the complete absence of DDAH1.

In the summary, our study demonstrated for the first time that chronic AGXT2 upregulation can protect from ADMA-mediated vascular injury in vivo without causing obvious phenotypical abnormalities. We have created a unique mouse model for studying the in vivo effects of ADMA lowering by a DDAH1-independent mechanism, which creates a basis for further exploration of AGXT2 as a possible target for cardiovascular protective interventions.

### Limitations

The transgenic AGXT2 mouse line, which we generated to assess chronic vascular benefits of ADMA lowering by a DDAH1-independent mechanism and the safety of chronic upregulation of AGXT2, overexpresses AGXT2 ubiquitously. It will have to be assessed in the future studies, whether tissue-specific overexpression of AGXT2 in its endogenous locations, i.e. in the liver and kidneys, would also lead to cardiovascular and/or metabolic benefits.

## Supplementary Information

Supplementary Figures.

## Data Availability

The datasets generated during and/or analyzed during the current study are available from the corresponding author on reasonable request.

## Abbreviations

ADGV: Asymmetric dimethylguanidino valeric acid
ADMA: Asymmetric dimethyarginine
DDAH1: Dimethylarginine dimethylaminohydrolase 1
DDAH2: Dimethylarginine dimethylaminohydrolase 2
SDGV: Symmetric dimethylguanidino valeric acid

## References

[1] Kielstein JT (2004). Cardiovascular effects of systemic nitric oxide synthase inhibition with asymmetrical dimethylarginine in humans. Circulation.

[2] Schlesinger S, Sonntag SR, Lieb W, Maas R (2016). Asymmetric and symmetric dimethylarginine as risk markers for total mortality and cardiovascular outcomes: A systematic review and meta-analysis of prospective studies. PLoS One.

[3] Leiper J (2007). Disruption of methylarginine metabolism impairs vascular homeostasis. Nat. Med..

[4] Hu X (2011). Dimethylarginine dimethylaminohydrolase-1 is the critical enzyme for degrading the cardiovascular risk factor asymmetrical dimethylarginine. Arterioscler. Thromb. Vasc. Biol..

[5] Dayoub H (2008). Overexpression of dimethylarginine dimethylaminohydrolase inhibits asymmetric dimethylarginine-induced endothelial dysfunction in the cerebral circulation. Stroke.

[6] Jacobi J (2010). Dimethylarginine dimethylaminohydrolase overexpression ameliorates atherosclerosis in apolipoprotein E-deficient mice by lowering asymmetric dimethylarginine. Am. J. Pathol..

[7] Vallance P, Leone A, Calver A, Collier J, Moncada S (1992). Accumulation of an endogenous inhibitor of nitric oxide synthesis in chronic renal failure. Lancet.

[8] Pope AJ, Karrupiah K, Kearns PN, Xia Y, Cardounel AJ (2009). Role of dimethylarginine dimethylaminohydrolases in the regulation of endothelial nitric oxide production. J. Biol. Chem..

[9] Jarzebska N, Mangoni AA, Martens-Lobenhoffer J, Bode-Boger SM, Rodionov RN (2019). The second life of methylarginines as cardiovascular targets. Int. J. Mol. Sci..

[10] Strobel J (2013). Transport of asymmetric dimethylarginine (ADMA) by cationic amino acid transporter 2 (CAT2), organic cation transporter 2 (OCT2) and multidrug and toxin extrusion protein 1 (MATE1). Amino Acids.

[11] Hasegawa K (2007). Role of asymmetric dimethylarginine in vascular injury in transgenic mice overexpressing dimethylarginie dimethylaminohydrolase 2. Circ. Res..

[12] Wang D (2007). Isoform-specific regulation by N(G), N(G)-dimethylarginine dimethylaminohydrolase of rat serum asymmetric dimethylarginine and vascular endothelium-derived relaxing factor/NO. Circ. Res..

[13] Schwedhelm E, Boger RH (2011). The role of asymmetric and symmetric dimethylarginines in renal disease. Nat. Rev. Nephrol..

[14] Dayoub H (2003). Dimethylarginine dimethylaminohydrolase regulates nitric oxide synthesis: Genetic and physiological evidence. Circulation.

[15] Liu X, Xu X, Shang R, Chen Y (2018). Asymmetric dimethylarginine (ADMA) as an important risk factor for the increased cardiovascular diseases and heart failure in chronic kidney disease. Nitric Oxide.

[16] Tain YL, Hsu CN (2017). Toxic dimethylarginines: Asymmetric dimethylarginine (ADMA) and symmetric dimethylarginine (SDMA). Toxins (Basel)..

[17] Willeit P (2015). Asymmetric dimethylarginine and cardiovascular risk: Systematic review and meta-analysis of 22 prospective studies. J. Am. Heart Assoc..

[18] Ghebremariam YT, Erlanson DA, Yamada K, Cooke JP (2012). Development of a dimethylarginine dimethylaminohydrolase (DDAH) assay for high-throughput chemical screening. J. Biomol. Screen.

[19] Hartzoulakis B (2007). Discovery of inhibitors of the pentein superfamily protein dimethylarginine dimethylaminohydrolase (DDAH), by virtual screening and hit analysis. Bioorg. Med. Chem. Lett..

[20] Linsky T, Fast W (2011). A continuous, fluorescent, high-throughput assay for human dimethylarginine dimethylaminohydrolase-1. J. Biomol. Screen.

[21] Caplin B (2012). Alanine-glyoxylate aminotransferase-2 metabolizes endogenous methylarginines, regulates NO, and controls blood pressure. Arterioscler. Thromb Vasc. Biol..

[22] Kittel A (2013). In vivo evidence that Agxt2 can regulate plasma levels of dimethylarginines in mice. Biochem. Biophys. Res. Commun..

[23] Rodionov RN, Murry DJ, Vaulman SF, Stevens JW, Lentz SR (2010). Human alanine-glyoxylate aminotransferase 2 lowers asymmetric dimethylarginine and protects from inhibition of nitric oxide production. J. Biol. Chem..

[24] Miyazaki J (1989). Expression vector system based on the chicken beta-actin promoter directs efficient production of interleukin-5. Gene.

[25] Martens-Lobenhoffer J, Bode-Boger SM (2012). Quantification of l-arginine, asymmetric dimethylarginine and symmetric dimethylarginine in human plasma: A step improvement in precision by stable isotope dilution mass spectrometry. J. Chromatogr. B Anal. Technol. Biomed. Life Sci..

[26] Martens-Lobenhoffer J, Rodionov RN, Drust A, Bode-Boger SM (2011). Detection and quantification of alpha-keto-delta-(N(G), N(G)-dimethylguanidino)valeric acid: A metabolite of asymmetric dimethylarginine. Anal. Biochem..

[27] Gu GH, Lim CK (1990). Separation of anionic and cationic compounds of biomedical interest by high-performance liquid chromatography on porous graphitic carbon. J. Chromatogr..

[28] Benz K (2018). Mild salt-sensitive hypertension in genetically determined low nephron number is associated with chloride but not sodium retention. Kidney Blood Press. Res..

[29] Jarzebska N (2019). Kidney and liver are the main organs of expression of a key metabolic enzyme alanine:glyoxylate aminotransferase 2 in humans. Atheroscler. Suppl..

[30] Langbein H (2016). NADPH oxidase 4 protects against development of endothelial dysfunction and atherosclerosis in LDL receptor deficient mice. Eur. Heart J..

[31] Hofmann A (2017). Lectin-like oxidized low-density lipoprotein receptor-1 promotes endothelial dysfunction in LDL receptor knockout background. Atheroscler. Suppl..

[32] Kobayashi M, Inoue K, Warabi E, Minami T, Kodama T (2005). A simple method of isolating mouse aortic endothelial cells. J. Atheroscler. Thromb..

[33] Newman PJ (1990). PECAM-1 (CD31) cloning and relation to adhesion molecules of the immunoglobulin gene superfamily. Science.

[34] Jacobi J (2005). Overexpression of dimethylarginine dimethylaminohydrolase reduces tissue asymmetric dimethylarginine levels and enhances angiogenesis. Circulation.

[35] Sydow K, Mondon CE, Schrader J, Konishi H, Cooke JP (2008). Dimethylarginine dimethylaminohydrolase overexpression enhances insulin sensitivity. Arterioscler. Thromb Vasc. Biol..

[36] Stuhlinger MC (2007). Asymmetric dimethyl l-arginine (ADMA) is a critical regulator of myocardial reperfusion injury. Cardiovasc. Res..

[37] Tanaka M (2005). Dimethylarginine dimethylaminohydrolase overexpression suppresses graft coronary artery disease. Circulation.

[38] Konishi H, Sydow K, Cooke JP (2007). Dimethylarginine dimethylaminohydrolase promotes endothelial repair after vascular injury. J. Am. Coll. Cardiol..

[39] Schwedhelm E (2009). Extensive characterization of the human DDAH1 transgenic mice. Pharmacol. Res..

[40] von Leitner EC (2011). Pathogenic cycle between the endogenous nitric oxide synthase inhibitor asymmetrical dimethylarginine and the leukocyte-derived hemoprotein myeloperoxidase. Circulation.

[41] Linsky T, Wang Y, Fast W (2011). Screening for dimethylarginine dimethylaminohydrolase inhibitors reveals ebselen as a bioavailable inactivator. ACS Med. Chem. Lett..

[42] Tokuo H (2001). Phosphorylation of neurofibromin by cAMP-dependent protein kinase is regulated via a cellular association of N(G), N(G)-dimethylarginine dimethylaminohydrolase. FEBS Lett..

[43] Li F (2006). Neurofibromin is a novel regulator of RAS-induced signals in primary vascular smooth muscle cells. Hum. Mol. Genet..

[44] Boult JK (2011). Active site mutant dimethylarginine dimethylaminohydrolase 1 expression confers an intermediate tumour phenotype in C6 gliomas. J. Pathol..

[45] Pullamsetti SS (2011). The role of dimethylarginine dimethylaminohydrolase in idiopathic pulmonary fibrosis. Sci. Transl. Med..

