# Supplementary material for: Overexpression of alanine-glyoxylate aminotransferase 2 protects from asymmetric dimethylarginine-induced endothelial dysfunction and aortic remodeling
Source: Sci Rep. 2022 Jun 7;12:9381. doi: 10.1038/s41598-022-13169-2 (PMC9174227; doi:10.1038/s41598-022-13169-2)
Supplement: Supplementary file 1 — Supplementary Figures. [file 41598_2022_13169_MOESM1_ESM.pdf]

Supplementary Material

Overexpression of alanine-glyoxylate aminotransferase 2 protects from asymmetric dimethylarginine-induced endothelial dysfunction and aortic remodeling

Roman N. Rodionov, Natalia Jarzebska, Dmitrii Burdin, Vladimir Todorov, Jens Martens-Lobenhoffer, Anja Hofmann, Anne Kolouschek, Nada Cordasic, Johannes Jacobi, Elena Rubets, Henning Morawietz, John O'Sullivan, Alexander G. Markov, Stefan R. Bornstein, Karl Hilgers, Renke Maas, Christian Pfluecke, YingJie Chen, Stefanie M. Bode-Böger, Christian P. M. Hugo, Bernd Hohenstein, Norbert Weiss

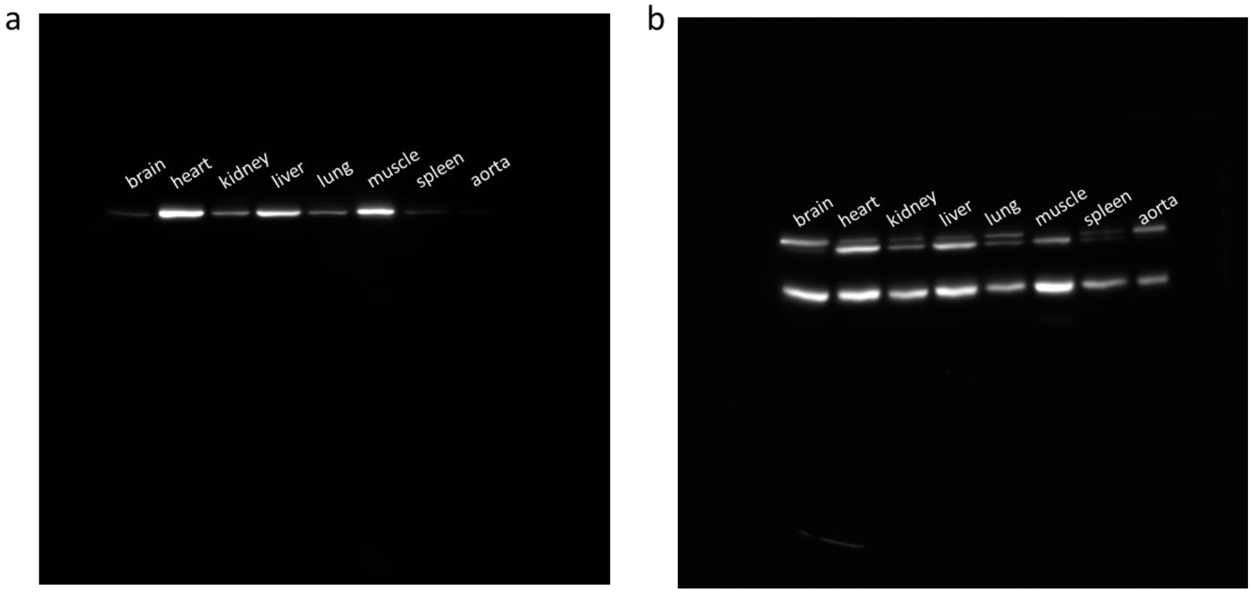

Supplementary Figure 1. Distribution of AGXT2 transgene in different tissues of TG mice, detected by Western blotting (unprocessed data). (a) AGXT2-FLAG, (b) GAPDH.

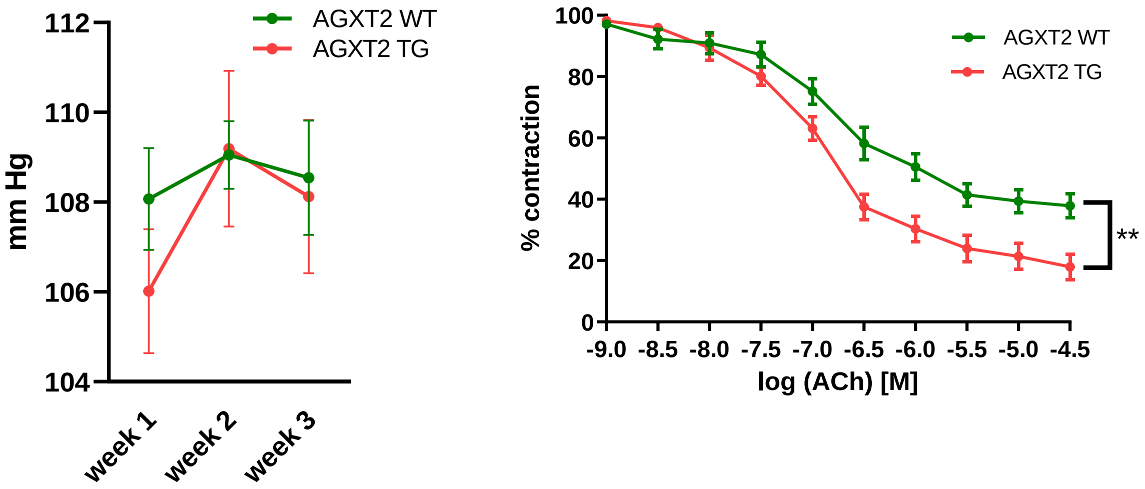

Supplementary Figure 2. Systolic blood pressure and endothelium-dependent vasodilation in AGXT2 WT and TG mice. repeated-measures ANOVA followed by Sidak multiple comparison test and two-way repeated-measures ANOVA followed by Sidak multiple comparison test, n=11-12. **p<0.01.
